# Evaluation of bioactive restorative materials on cell viability using direct and extract methods

**DOI:** 10.1590/1807-3107bor-2026.vol40.017

**Published:** 2026-03-30

**Authors:** Zeynep Ekin KILINC, Vahdi Umut BENGI, Selcuk SAVAS, Ebru KUCUKYILMAZ

**Affiliations:** (a) Canpol Dental Clinic, Pediatric Dentistry, Eskisehir, Turkey.; (b) Gulhane Health Sciences University, Gulhane School of Dentistry, Department of Periodontics, Ankara, Turkey.; (c) Izmir Katip Celebi University, School of Dentistry, Department of Pediatric Dentistry, Izmir, Turkey,

**Keywords:** Cell Surviveal, Composite Resins, Compomers

## Abstract

The biological response of gingival fibroblasts to restorative materials is a key factor in determining the clinical success. This study aimed to evaluate the effects of four restorative materials on the viability of gingival fibroblast cell cultures using a real-time cell analysis system with direct extract methods. Four different restorative materials with bioactive properties were used in this study: Glasiosite (VOCO GmbH, Cuxhaven, Germany), BEAUTIFIL-Bulk Restorative (Shofu Inc., Kyoto, Japan), EQUIA Forte™ HT Fil (GC Corporation, Tokyo, Japan), and Activa BioACTIVE Restorative® (Pulpdent Corporation, Watertown, USA). Disc-shaped specimens were prepared for each material group (n = 18 and n = 9 for each test method). The effects of the materials on gingival fibroblast viability were determined using both direct and extract methods with a real-time cell analysis system (xCELLigence) at two different time periods (24 h and 48 h). A significance level of α = 0.05 was adopted for all statistical analyses. The control group exhibited the highest cell viability, and the differences between the groups were statistically significant at both 24 h and 48 h (p < 0.05). At both 24 h and 48 h, Glasiosite showed the highest cell viability among the tested materials, whereas the BEAUTIFIL-Bulk Restorative exhibited the lowest cell viability (p < 0.05). Cell viability was significantly higher with the extract method than with the direct contact method across all materials, except for the control (p < 0.05). This study revealed that the cell viability varied significantly depending on the material type, exposure time, and test method. Glasiosite showed the highest biocompatibility, while the BEAUTIFIL-Bulk Restorative exhibited the lowest value.

## Introduction

The primary objective of pediatric restorative treatment is to eliminate cavitation, structural defects, and demineralization processes occurring on tooth surfaces and preserve the dental tissue integrity. In contemporary dentistry, the development of restorative materials remains a key area of research.^1,2^ Newly developed materials are expected to fulfil esthetic demands, demonstrate superior physical and mechanical properties, promote remineralization of dental tissues, and maintain high biocompatibility without eliciting cytotoxic or inflammatory responses in the surrounding tissues. Although these are regarded as ideal characteristics of restorative materials, none of the currently available products can fulfill all these features simultaneously, and the search for alternative solutions is ongoing.

Recently, bioactive materials developed for restorative purposes have attracted increasing attention in dentistry because of their ability to contribute to the structure and chemistry of natural tooth tissue. Among these materials, EQUIA Forte™ HT Fil is a high viscosity glass ionomer restorative material, that exhibits superior physicomechanical properties compared to conventional glass ionomer cements.^2,3^It has a higher fluoride release ability compared to conventional glass ionomer cements, with cumulative values reported to be approximately two to four times greater.^4^ Its optimized particle size and distribution provide enhanced mechanical strength, crack resistance, and marginal stability.^3,5,6^ The decreased refractive index of the glass provides a better match with the matrix, resulting in higher translucency and improved esthetics.^6,7^ This material, when used in combination with its specific surface coating agent, allows to obtain smooth and glossy surface finishes.^5^

Although glass ionomer-based materials have attracted attention owing to their biologically active properties, studies are still ongoing to develop alternative materials with improved mechanical properties while maintaining this biological activity.^8-10^ In this context, the aim is to prevent demineralization and support remineralization with the use of compomers, resin-modified glass ionomers, and new-generation resin-based restorative materials with modified compositions to increase the ion release capacity.^8-15^ In the literature, these materials are reported to offer superior mechanical properties, such as higher tensile and flexural strengths and increased wear resistance, compared to conventional glass ionomer cements.^8,10,12^ Such improvements in durability may lead to longer ion release, which in turn could enhance their anticariogenic potential.

Among newly developed materials, ACTIVA BioACTIVE^®^ Restorative is a resin based bioactive material designed to facilitate ion exchange, mimicking the natural behavior of teeth. Unlike conventional resin-based materials, which are inert, Activa BioACTIVE Restorative® has an ion-releasing resin matrix that allows calcium, phosphate, and fluoride ions to be released and recharged.^9,10^Combining the mechanical strength and longevity of resin-based materials with the bioactive properties of glass ionomers, this material stands out as a remarkable restorative material alternative because of its potential remineralization capacity as well as its improved moisture resistance and fracture toughness.^9-12^

Another group of restorative materials that has been introduced in recent years is bulk-fill resin composites with bioactive properties, such as those containing surface pre-reacted glass (S-PRG) fillers (commercially referred to as “Giomers” by the manufacturer). These materials contain pre-reacted glass ionomer (PRG) particles, which have the capacity to release ions that promote remineralization, including fluoride, sodium, borate, aluminium, silicate, and strontium. Moreover, a further benefit of this technology is its ion reloadability.^13,14^ It has been documented that ion release levels in these S-PRG–containing composites are elevated in comparison to those observed in compomers and composite resins.^15^ Beyond their chemical properties, such materials are also notable for their favorable physical performance, ease of use in clinical applications, and positive treatment outcomes.^16^ Considering these features, the clinical preference for S-PRG–containing bioactive composites has been steadily increasing. However, it is noteworthy that the number of studies evaluating these materials in the current literature remains limited.

A high level of clinical success can be achieved if restorative materials are selected according to appropriate indications and applied using meticulous clinical techniques and correct protocols. Cavities located at or extending apically from the cemento-enamel junction (CEJ) are frequently encountered in clinical practice, and present unique restorative challenges due to anatomical and technical constraints.^17^ In particular, the direct contact of restorative materials with gingival tissues has the potential to compromise biological integrity, thus necessitating greater caution in material selection. Human gingival fibroblast cells are pivotal in preserving the physiological balance of the periodontal tissues, through the mediation of vital biological processes including the production of the extracellular matrix, regulation of inflammatory responses, and facilitation of wound healing.^18^ In this regard, an assessment of the effects of restorative materials on these cells offers valuable insight into the clinical performance and long-term biocompatibility of the materials in periodontal environments.^19,20^ Previous *in vitro* studies have demonstrated that conventional and resin-modified glass ionomers can exhibit significant cytotoxicity compared to control groups, and particularly in the short term, they may induce adverse cellular responses in gingival fibroblasts.^20-22^ Furthermore, resin-based restorative materials may display variable cytotoxic effects related to residual monomer release, while compomers and bioactive resin systems have been associated with lower cytotoxicity compared to conventional composites.^23-28^ The effects of S-PRG–containing bioactive composites on fibroblast viability, however, remain insufficiently explored.^29^Collectively, these data suggest that restorative materials with different compositions may elicit distinct biological responses, thereby justifying further comparative investigations.

Therefore, the objective of this *in vitro* study was to comparatively evaluate the potential biological effects of four bioactive restorative materials on the viability of gingival fibroblast cell cultures using a real-time cell analysis system with direct and extract methods. In line with the objectives of this study, the hypotheses were as follows: (1) the type of material significantly affects the viability of human gingival fibroblast cells depending on the testing method employed; (2) there is a significant difference between the viability of gingival fibroblast cells incubated on each material for different periods (24 h vs. 48 h), irrespective of the testing method; and (3) there is a significant difference in cell viability outcomes between the direct and extract testing methods.

## Methods

**Figure 1:**
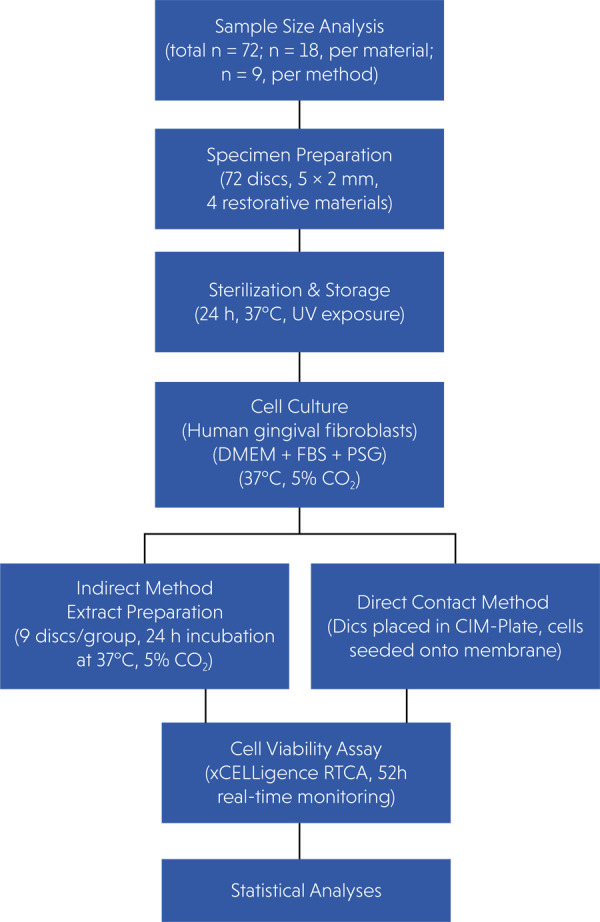
Flowchart of the study.

The study protocol was approved by the Izmir Katip Celebi University Health Research Ethics Committee (approval no.: 2021/*0*233). Prior to the study, a sample size analysis was performed (G*POWER 3.1.9 Power Analysis Sample Size Software) with an effect size of f = 0.35, a confidence interval (CI) of 0.05, and a power level of 90% for Type I error. The sample size was estimated to be 9 for each group for each test. A total of 72-disc shaped samples (half of each group was tested using either the direct contact (n = 36) or extract (n = 36) methods) were included in the study. Four restorative materials with different compositions were used in the study: Glasiosite, Fuji Triage^®^, EQUIA Forte^™^ HT Fil, BEAUTIFIL-Bulk Restorative, and Activa BioACTIVE Restorative®. Two different treatment periods (24 and 48 h) were evaluated. The chemical compositions, manufacturers, batch numbers, and application methods of the tested materials are listed in Table 1. A flowchart of the study is shown in Figure 1.

**Table 1:** Chemical composition, material type and manufacturers of the tested materials.

Materials	Chemical composition	Material type	Batch numbers	Manufacturer	Application procedure
Glasiosite	BIS-GMA, UDMA, TEGDMA, BHT, Filler 77.5 wt% (SiO2, AlSi, FAlSi)	Compomer	#1937604	VOCO GmbH, Cuxhaven, Germany	Dispense the material directly into the specimen mold. Light cure both sides of the specimen for 20 s using an LED curing light.
BEAUTIFIL-Bulk Restorative	Bis-GMA, UDMA, Bis-MPEPP, TEGDMA, S-PRG filler based on fluoroboroaluminosilicate glass, polymerization initiator, pigments, and others	Bulk-fill resin composite	#081938	Shofu Inc., Kyoto, Japan	Insert the material directly into the specimen mold. Light cure both sides of the specimen for 20 s using an LED curing light.
EQUIA Forte^™^ HT Fil	Powder: strontium fluoroalumino-silicate glass (including highly reactive small particles), polyacrylic acid powder liquid: polyacrylic acid, polycarboxylic acid, tartaric acid	High-viscosity conventional glass ionomer cement	#2005191	GC Corporation, Tokyo, Japan	Shake the capsule and mix it in a capsule mixer for 10 s. Immediately dispense the material into the specimen mold and allow it to set.
ACTIVA BioACTIVE Restorative®	Diurethane and methacrylates with modified polyacrylic acid (44.6%), reactive glass filler (21.8 wt%), inorganic filler (56 wt%), patented rubberized resin (Embrace), water	Resin with modified glass filler	#190110	Pulpdent Corporation, Waterdown, USA	Dispense the material into the specimen mold. Light cure both sides of the specimen for 20 s using an LED curing light.

*BisGMA: Bisfenol diglisidilmethacrylate; BisEMA: bisfenolmetilmethacrylate; UDMA: uretan dimethacrylate; PEGDMA: polietilen glikol dimethacrylate; TEGDMA: Triethylene glycol dimethacrylate; Bis-MEPP: 2,2-bis (4-methacryloxypolyethoxyphenyl) propane.

### Specimen preparation

#### Preparation of restorative material specimens

A total of 72 specimens (18 specimens for each restorative material, n = 9 for each method) were prepared using a specially designed metal mold (5.0 mm in diameter × 2 mm in depth).^22, 30^ The specimens were prepared following a standardized method, in which the material was pressed into the mold between two glass slides covered with Mylar strips (Hawe™ StopStrip Brea, California). All materials were used in accordance with the manufacturers’ instructions (Table 1). The surfaces of each specimen were light-cured on both sides using an LED curing light in standardized mode (Valo Cordless LED, Valo, Ultradent, St Louis, USA) operating at an intensity of 1,000 mW/cm^2^. The curing light intensity was checked periodically for every five specimens using a radiometer (Radiometer 100, Demetron Research Corp., Danbury, USA). All specimens were first stored in a humid environment at 37°C 5% CO₂ (Galaxy 170S CO₂ Incubator, Eppendorf, Framingham, USA) for 24 h to allow for complete polymerization, and subsequently sterilized by exposure to ultraviolet light (Light Sources Inc., Orange, , USA), first for 1 h on one surface and then for another 1 h on the opposite surface.^17^No polishing procedures were applied to the specimens in order to preserve their standardization.

## Gingival fibroblast cell culture

Primary human gingival fibroblasts (hGFs) were isolated from the gingival tissue of a healthy donor at the Department of Periodontology, Gulhane Faculty of Dentistry, University of Health Sciences, Ankara, Turkey. Gingival fibroblast cells were cultured in Dulbecco’s Modified Eagle Medium (DMEM) (Pan Biotech, Aidenbach, Germany) supplemented with 10% fetal bovine serum (FBS) (Capricorn Scientific GmbH, Ebsdorfergrund, Germany) and 1% penicillin-streptomycin-glutamine (PSG) (Sigma-Aldrich, St. Louis, USA).^31^The cultures were maintained in a humidified atmosphere of 5% CO₂ at 37°C (Galaxy 170S CO₂ Incubator, Eppendorf, Framingham, USA), and the culture medium was refreshed every 48 h to ensure optimal cell proliferation and viability. When the cells covered the bottom surface of the 35 cm^2^ culture flasks, the culture medium was aspirated and the cells were gently washed by pipetting with DMEM. The cells were detached by treating with 0.25% trypsin–0.21 mM EDTA solution for 5 min at 37°C. Following microscopic confirmation of cell detachment (Zeiss Stemi 2000-C and Discovery V8 Stereo Microscopes, Carl Zeiss Microimaging GmbH, Göttingen, Germany), the cell suspension was collected using sterile 2 mL serological pipettes and transferred into capped centrifuge tubes. The cells were centrifuged at 200 rpm for 5 min (NUVE NF1200, Ankara, Turkey) to pellet the cells and remove the EDTA and trypsin solution. After centrifugation, the supernatant was discarded and the cell pellet was resuspended in fresh DMEM containing 10% FBS and 1% PSG. This resuspension process was repeated five times to ensure the complete removal of residual trypsin-EDTA and promote a homogeneous cell suspension. The cells were counted using an automatic cell counter (TC20 Automated Cell Counter; Bio-Rad, Dubai, United Arab Emirates). The mean cell density obtained from two independent counts was 11,600 cells/ml.

## Cell viability testing

The cells were seeded into E-Plates (ACEA Biosciences Inc., San Diego, CA, USA) for the extract method, and CIM-Plates (Agilent, San Diego, USA) for the migration and invasion steps in the direct method for the real-time cell analysis system. Each well was filled with 150 μL of culture medium and then incubated at 37°C with 5% CO₂ for 48 h. After counting the prepared cells, a total of 2.3 × 10⁴ cells were seeded into each well. After seeding, the plates were incubated for 48 h at 37°C under 5% CO₂ to allow for optimal cell adhesion and stabilization before the experimental interventions. In addition, wells containing only the culture medium served as a negative control, whereas wells containing PBS-supplemented medium were included as an additional reference group.

## Preparation of material extracts (indirect contact method)

Nine discs were allocated to each material group and placed in sterile Eppendorf tubes. Subsequently, a total of 1.2 mL of freshly prepared culture medium was prepared per group (n = 9) and calculated according to the ISO 10993-5 recommended extraction ratio of 5 cm^2^/mL. The specimens were incubated in a humidified environment at 37°C with 5% CO₂ for 24 h to allow the leaching of soluble components into the medium without any direct contact with the cells. Following the incubation period, 100 μL aliquots of the resulting extracts were aseptically transferred into the wells of E-Plate (ACEA Biosciences, San Diego, USA) using sterile pipettes for real-time cytotoxicity analysis. The E-Plate was monitored for 52 h with hourly measurements using a real-time cell analysis system, and the cell viability was recorded.

## Direct contact method

For the direct contact assessment, 200 µL of DMEM was initially added to the lower chambers of the CIM-Plate and pre-incubated for 30 min at 37°C with 5% CO₂ to equilibrate the system. The test material discs were then carefully placed onto the upper membrane inserts of the CIM-Plate, which were covered with the lid of the CIM-Plate and incubated again for 30 min under the same conditions. Subsequently, the upper chambers were filled with cell suspensions at a density of 2.0 × 10⁴ cells per well. Similar to the extract method, wells containing PBS-supplemented media served as an additional reference group, whereas wells containing only the medium served as negative controls. After assembly, the plates were inserted into the xCELLigence RTCA DP instrument (Agilent, Santa Clara, USA), cell index values were automatically recorded every hour over a 52-h period, and cell viability was assessed and recorded.

## Statistical analyses

Statistical analyses were performed using the SPSS software (version 25, IBM, Chicago, USA). Shapiro–Wilk test was used to assess the normality of data distribution, and Levene’s test was conducted to evaluate the homogeneity of variances. Because the assumptions for parametric testing were not satisfied, non-parametric tests were applied. Comparisons between different time points (24 h vs. 48 h) within the same material group were performed using Wilcoxon signed-rank tests. Comparisons between different material groups at the same time point were conducted using Kruskal–Wallis test, followed by Mann–Whitney U test for post-hoc analyses. Data are presented as medians and quartiles for each subgroup. A significance level of α = 0.05 was adopted for all statistical analyses.

## Results

**Figure 2:**
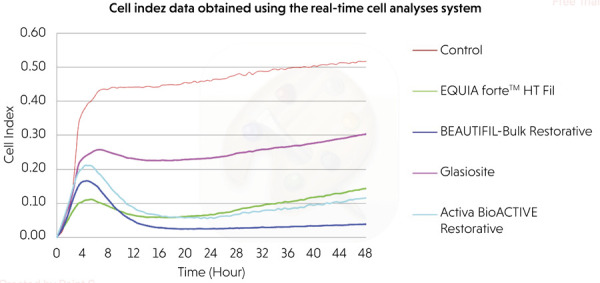
Evaluation of cell viability using the direct contact method with a real-time cell analysis system.

**Figure 3:**
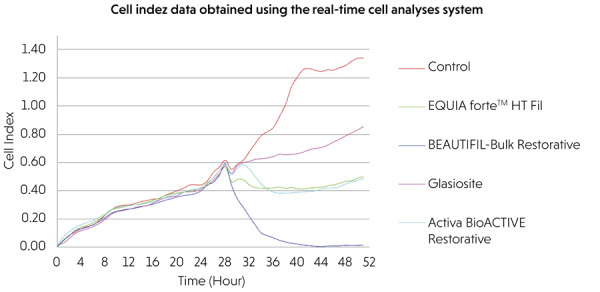
Evaluation of cell viability using the extract method with a real-time cell analysis system.

The findings obtained from the comparison of each material group at two different time points (24 h and 48 h) using two different methods (extract and direct contact) with the real-time cell analysis system are presented in Table 2 and Figures 2 and 3.

**Table 2:** Evaluation of data for material groups assessed for cell viability using real-time cell analysis system with direct contact and extract methods (Median [Q1–Q3]).

Groups	Direct contact method		Direct contact method		p-value*	Extract method		Extract method		p-value*
	24 hours	% Cell viability	48 hours	% Cell viability		24 hours	% Cell viability	48 hours	% Cell viability	
Glasiosite	0.23^a, A^	50.5	0.30^a, X^	58	< 0,001	0.28α^, B^	90.3	0.60α^, Y^	87	< 0,001
	(0.23-0.24)		(0.29-0.31)			(0.25-0.30)		(0.57-0.63)		
BEAUTIFIL-Bulk Restorative	0.02^b, A^	5.4	0.03^b, X^	7.4	< 0,001	0.27α^, B^	87.1	0.25β^, Y^	36.2	0.489
	(0.02-0.03)		(0.03-0.04)			(0.26-0.30)		(0.08-0.39)		
EQUIA Forte^™^ HT Fil	0.06^c, A^	14.8	0.14^c, X^	27.5	< 0,001	0.30α^, B^	96.8	0.45γ^, Y^	65.2	< 0,001
	(0.06-0.07)		(0.13-0.14)			(0.28-0.33)		(0.41-0.47)		
Activa BioACTIVE Restorative^®^	0.05^c, A^	12.7	0.11^c, X^	22.2	< 0,001	0.29α^, B^	93.5	0.50γ^, Y^	72.5	< 0,001
	(0.05-0.06)		(0.11-0.12)			(0.28-0.32)		(0.42-0.57)		
Control	0.46^d, A^	100	0.51^d, X^	100	< 0,001	0.31α^, B^	100	0.69α^, Y^	100	< 0,001
	(0.46-0.46)		(0.51-0.52)			(0.29-0.34)		(0.60-0.80)		
p-value**	< 0,001		< 0,001			0,066		< 0,001		

*Wilcoxon signed rank test; ** Kruskal Wallis-post hoc Man Whitney U test. Different lowercase superscript letters (a, b, c, d) indicate statistically significant differences among material groups at the 24- and 48-hour evaluation using the direct method. *Different lowercase superscript letters (α, β, γ) indicate statistically significant differences among material groups at the 24- and 48-hour evaluation using the extract method. Different uppercase superscript letters (A, B) indicate statistically significant differences between the extract and direct methods at the 24-hour evaluation. *X, Y: Indicate statistically significant differences between the extract and direct methods regarding cell viability values at the 48-hour evaluation.

### Evaluation of cell viability by direct method

In the direct contact method, when the material groups were compared at each time points (24 and 48 h), the control group exhibited the highest cell viability. The differences between the groups were statistically significant at both 24 and 48 h (p < 0.001 for both). Among the tested materials, the ranking of cell viability at both the time points was as follows: Glasiosite > EQUIA Forte™ HT Fil > Activa BioACTIVE Restorative^®^ > BEAUTIFIL-Bulk Restorative. Within-group comparisons across time points (24 vs. 48 h) for each material in the direct method revealed that the 48-h value was higher than that at 24-h (all p 0.001).

### Evaluation of cell viability by extract method

In the extract method, no statistically significant differences were observed among the material groups at 24 h (p = 0.066), whereas significant differences were observed at 48 h (p < 0.001). The control group exhibited the highest cell index values at both the time points. Among the materials, Glasiosite demonstrated the highest, followed by Activa BioACTIVE Restorative^®^ and EQUIA Forte™ HT Fil, with BEAUTIFIL-Bulk Restorative showing the lowest values. Within-group comparisons indicated a statistically significant increase in cell index values from 24 h to 48 h for Glasiosite, Activa BioACTIVE Restorative^®^, EQUIA Forte™ HT Fil, and the control group (p < 0.001), while no significant change was detected for BEAUTIFIL-Bulk Restorative (p = 0.489).

At 24 h cell viability ranked as control (100%) > Glasiosite (50.5%) > EQUIA Forte (14.8%) ≈ Activa (12.7%) > Beautifil-Bulk (5.4%). At 48 h, the order was control (100%) > Glasiosite (58%) > EQUIA Forte (27.5%) > Activa (22.2%) > Beautifil-Bulk (7.4%).

### Comparison of cell viability between extract and direct contact methods

Statistically significant differences in cell index values were observed between the direct and extract methods for all material groups evaluated at both 24 and 48 h (p < 0.005). Across all materials, the extract method resulted in greater cell proliferation than the direct method, except for the control groups.

At 24 h, the viability order was as follows: control (100%) > EQUIA Forte (96.8%) > Activa (93.5%) > Glasiosite (90.3%) > Beautifil-Bulk (87.1%). After 48 h, the order was as follows: control (100%) > Glasiosite (87%) > Activa (72.5%) > EQUIA Forte (65.2%) > Beautifil-Bulk (36.2%).

## Discussion

Examination of the potential cytotoxic effects of new-generation materials and the objective evaluation of their biocompatibility have gained importance both clinically and scientifically. This study, therefore, sought to evaluate the cytotoxic effects of four different bioactive restorative materials, namely Glasiosite, BEAUTIFIL-Bulk Restorative, EQUIA Forte™ HT Fil, and ACTIVA BioACTIVE Restorative®, on human gingival fibroblast cells. The evaluation was conducted at 24 and 48 h using the direct contact and extract methods to compare the effects of the materials on cell viability and determine their biological compatibility.

Human fibroblast cells are widely used and recommended in cytocompatibility studies, as they can be more easily cultured, maintained, and standardized compared to epithelial cells.^18-20^ Although epithelial cells are in direct contact with class V restorations, fibroblasts provide crucial information about the response of the underlying connective tissue, thereby offering a reliable and comparable model in line with previous studies.^18-20^ This study employed the iCELLigence® system (an impedance-based real-time cell analysis system) to evaluate the cytotoxic effects of dental materials at the cellular level with greater accuracy and dynamism.^23, 24^ Real-time analysis systems allow the measurement of cell index values through many time points and create a line graph that reflects the biological status of the cells. Continuous quantitative readout of cell viability helps obtain more realistic results compared to single end-point values of conventional cytotoxicity tests.^23,24^ In this study, two different exposure periods (24 and 48 h) were selected to evaluate the cytotoxic effects of the tested restorative materials on gingival fibroblasts. This choice is also supported by previous *in vitro* investigations.^22,23^ Accordingly, the 24 h period provides information on the initial interaction between the cells and the components released from the material, whereas the 48 h period allows for a clearer observation of the changes occurring within the same interval following this initial interaction.^22,23^The evaluation of the cytotoxic effects of dental materials is permitted by three fundamental *in vitro* methods: direct contact, indirect contact, and extract methods.^18-21,32^The direct contact method is predicated on the direct contact of the test material with the cell monolayer. This method offers significant advantages in mimicking clinical scenarios where the material is in direct contact with the oral soft tissues.^18,21^ The extract method involves the incubation of the material in a suitable liquid, such as a cell culture medium, for a designated period, followed by contact of the extract obtained from this medium with the cells.^32,33^ It is important to note that in clinical applications, restorative materials are not always in direct contact with soft tissue. Therefore, this extract method has the potential for use in many situations. Therefore, in this study, human gingival fibroblast cells were employed together with a real-time analysis system, and cytotoxicity was assessed dynamically at 24- and 48-h intervals using both direct contact and extract methods.

Based on the findings of this study, the following hypotheses were accepted because of the presence of statistically significant differences: a) the type of restorative material would affect the viability of gingival fibroblast cells regardless of the testing method employed; b) the incubation time would have a significant influence on cell viability irrespective of the testing method; and c) there would be a significant difference in cell viability between the direct and extract testing methods.

Using the direct contact method, Glasiosite demonstrated the highest cell viability, although it remained significantly lower than that of the control group. In contrast, the Beautifil-Bulk Restorative exhibited the lowest viability at both the time points. In contrast, cell viability values were generally higher for Glasiosite, Activa, and EQUIA Forte in the extract method, suggesting that indirect exposure reduced the cytotoxic effects of the released components. Notably, the Beautifil-Bulk Restorative exhibited a significant decrease in viability after 48 h under the extract conditions, indicating the continuous release of cytotoxic components. These results highlight the significant impact of the material composition and test method on biocompatibility outcomes.

Glasiosite exhibited the highest levels of cell viability in both the testing methods and at both the time points compared with the other tested materials. However, there is a lack of directly comparable studies on the cytotoxic profile of this specific product. Nevertheless, several studies have demonstrated that compomer group materials typically exhibit reduced levels of toxicity in comparison to resin composites and resin modified glass ionomer group restorative materials.^25-28^ Chen et al. reported that the cytotoxic effects of compomer materials on human deciduous tooth pulp cells were significantly lower than those of composites and resin modified glass ionomers.^25^ Schweikl et al. reported that compomers released less cytotoxic monomers and exhibited reduced polymerization shrinkage in comparison to conventional composite resins.^26^Moreover, as stated by Botsali et al. in their study on fibroblast attachment, the utilization of compomers resulted in a higher level of cell attachment in comparison to alternative materials.^28^ The authors stated that his phenomenon may be attributed to the surface characteristics of the material. Although Glasiosite also contains monomers with potential cytotoxic effects, the relatively lower cytotoxicity observed in this study may be attributed to its reduced resin content, matrix formulation, and physicochemical properties, which could limit the extent of monomer release and cellular impact. The findings of this study are consistent with those of similar reports in the literature and indicate that although Glasiosite resulted in a cell viability below the cytotoxicity threshold in the direct method, it still demonstrates a biocompatibility potential superior to the other tested materials in terms of preserving cell viability.

Beautifil Bulk had the lowest cell viability at all time points among the materials tested in this study. This cytotoxic effect is thought to be caused by the release of toxic monomers such as TEGDMA (triethylene glycol dimethacrylate) and Bis-GMA (bisphenol-A glycidyl methacrylate) in the structure of this material and ions released from PRG fillers.^13,34^ Bis-GMA and TEGDMA have been reported to show harmful effects on various cell lines such as human gingival cells, pulpal fibroblasts, monocytes, and erythrocytes.^27,28,35^Due to its high molecular weight and hydrophobic structure, Bis-GMA can leak into the surrounding tissues as a free monomer when polymerisation is not complete.^32,33^ This monomer integrates into fibroblast cell membrane, increases membrane permeability, disrupts ion balance, and affects intracellular calcium homeostasis.^36,37^ As a result, mitochondrial membrane potential decreases, oxidative stress increases, and reactive oxygen species (ROS) accumulate. ROS accumulation leads to oxidative damage in DNA, protein and lipid structures.^34,36,37^ Furthermore, Bis-GMA can cause cytotoxicity not only through apoptosis but also through processes such as necrosis, cell cycle arrest, and pro-inflammatory cytokine expression (e.g. IL-6 and TNF-α).^34,36,37^ These effects lead to reduced fibroblast proliferation, impaired collagen production, and delayed tissue regeneration.^34^ On the other hand, TEGDMA, which is added to the organic phase of resin-based materials to adjust the viscosity, can easily pass into biological compartments due to its low molecular weight and high hydrophilic structure. This property causes TEGDMA to penetrate many cellular sites, including the cell nucleus, affecting physiological processes, such as cell growth and differentiation.^28,36^ Furthermore, TEGDMA can induce cellular stress by generating ROS depending on the time and dose, which can result in apoptosis or necrosis.^28,36^In addition to monomer release, various ions such as fluoride, aluminum, boron, sodium, silicon, silicon, strontium, and zinc in the structure of S-PRG-containing bioactive materials can also cause potential toxic effects on the surrounding tissue. It is stated that fluoride may cause tissue toxicity at high concentrations through mechanisms, such as enzyme inhibition, oxidative stress, inflammation, and apoptosis.^38-40^ Several studies have shown that monomer release in S-PRG–containing bioactive materials continues even days after polymerization, leading to prolonged cytotoxic effects.^38-40^ Toh et al. reported that Beautifil Bulk Restorative had the highest level of cytotoxic activity among the available bulk-fill composites and that this material did not show a significant improvement in cell viability even after 48 h.^29^ These findings suggest that the cytotoxicity of Beautifil Bulk Restorative is influenced by both monomer release and ionic component-related mechanisms, which is consistent with the cell viability data obtained in our study.

In this study, Activa BioACTIVE Restorative^®^ and EQUIA Forte™ HT Fil materials exhibited similar cytotoxicity profiles at all experimental time points and in both the test methods applied. Although no significant difference was found between the two materials, both showed greater cytotoxicity than the control, lower cytotoxicity than the Beautifil Bulk Restorative, and higher cytotoxicity than Glasiosite. These findings suggest that although these materials may exhibit some degree of biological activity through ion release, they do not provide a notable advantage in supporting fibroblast viability. Activa BioACTIVE Restorative® is an innovative bioactive restorative material that, although in the resin class, differs from traditional composite materials in terms of content and biological interaction. The material does not contain BisGMA, which is controversial because of its endocrine-disrupting effects. Instead, it contains biocompatible glass particles and a moisture-sensitive resin matrix.^6-9^ This structure allows chemical bonding to the tooth tissue and marginal sealing by forming an apatite-like surface layer. In addition, it aims to mimic the natural tooth tissue both chemically and biologically because of its ability to simultaneously release calcium, phosphate, and fluoride ions.^6-9^

Bioactive materials promote the release of Ca2+ ions that have mitogenic effects on cells. In agreement with the present findings, López-García et al. reported significantly better biocompatibility, cell adhesion, migration, and morphology for Activa BioACTIVE Restorative® than for other conventional glass ionomer-based materials they tested. They suggested that calcium and phosphorus released as a result of degradation of the bioactive glass in Activa BioACTIVE Restorative® might have favorable effects on cell viability and proliferation. In addition, the absence of HEMA (hidroksietilmetakrilat), with its established cytotoxicity effect, in Activa BioACTIVE Restorative® could have accounted for the latter’s better biocompatibility relative to RMGI (resin modified glass ionomer). It has been reported that cell irritation and cytotoxicity of RMGI are due to its high HEMA content. EQUIA Forte™ HT Fil is a high viscosity glass ionomer reinforced with ultrafine glass particles. These highly reactive glass structures have been developed to improve the mechanical and biological performances of materials. However, the polyalkenoic acid contained in the material may adversely affect cellular viability by causing a local pH drop and high levels of fluorine release in cases where the buffering capacity is insufficient.^39^ High fluoride release observed especially in the first 24 h may suppress cell growth, proliferation, and protein synthesis.^41^ In addition, the relatively high surface roughness of glass ionomer-based materials compared to resin-based materials (e.g., compomers) limits the adhesion and invasion of oral cells, which is consistent with studies showing an inverse relationship between fibroblast viability and surface roughness.^42, 43^

There are two main studies in the literature comparing the cytotoxic effects of Activa BioACTIVE Restorative® and EQUIA Forte™ HT Fil.^19, 21^ In the first of these studies, both materials were evaluated together with Fuji II LC (resin modified glass ionomer) and Tetric EvoFlow (bulk-fill composite), and it was reported that all materials showed cytotoxic effects compared to the control group at 0-72 h. However, an increase in the cell viability rates for all materials was observed at the end of 72 h, suggesting that these materials may develop biological tolerance over time. In the study, Activa BioACTIVE was reported to provide slightly higher cell viability compared to EQUIA Forte™ HT Fil.^19^In the real-time cytotoxicity analysis performed by Kolus et al., it was reported that both the materials showed cytotoxic effect in undiluted extracts in tests performed with L929 fibroblast cells; however, this effect was significantly reduced in diluted conditions. It was also reported that Activa BioACTIVE Restorative® and EQUIA Forte™ HT Fil showed similar results in terms of cell viability and that both materials offered a better biocompatibility profile compared to conventional glass ionomers.^21^ In a study conducted with dental pulp stem cells, López-García et al. also reported that Activa BioACTIVE showed low ROS production and high proliferation potential, positively affected cell morphology, increased cell migration and adhesion, and provided higher cell attachment, especially compared to conventional glass ionomer-based materials. However, they also emphasized that the resin matrix it contains may partially limit these positive biological effects by releasing reactive monomers during the early polymerization process.^23^ On the other hand, it is also emphasized in the literature that EQUIA Forte™ HT Fil, apart from its high initial fluorine release, supports cell proliferation and offers an acceptable level of biological tolerance, especially under diluted extract conditions.^41^

In this study, a significantly lower cell viability was observed for all materials in the direct-contact method than in the extract method. As mentioned earlier, in the direct method, cells are directly exposed to substances released from the material surfaces, surface irregularities, and pH changes. In contrast, the extract method indirectly affects the cells, simulating the dilution effect of saliva *in vivo*. It has been reported in the literature that residual monomers such as HEMA, TEGDMA, and UDMA (urethan dimetakrilat) from resin-based materials diffuse within the first 24-72 h after polymerization and may cause cytotoxic, genotoxic, and pro-inflammatory responses.^28^ This was particularly evident in the BEAUTIFIL-Bulk Restorative group and consistently low cell viability values were recorded in both methods. Importantly, within the first 48 h, none of the materials tested using the direct contact method exceeded the acceptable threshold for cytotoxicity. However, no significant difference was found between the material groups in the 24-h evaluations performed using the extract method. This indicated that early release under indirect exposure conditions may not always cause detectable toxicity. This reveals the importance of using both test methods for a comprehensive evaluation of material biocompatibility.

Time-series data revealed an overall increase in cell viability after 48 h. This increase may be related to a decrease in residual substances as the materials undergo post-curing stabilization and the cells adapt to their surroundings. However, this trend did not apply to the BEAUTIFIL-Bulk Restorative, which maintained its toxic effects. This is consistent with studies indicating that monomer-induced oxidative stress is not necessarily transient and has potential long-term effects, particularly when materials exhibit a sustained-release profile.^35^

Although in vitro studies do not provide data as strong as clinical studies, they allow targeted evaluations by creating environmental conditions under which various factors are stabilized. This study had some limitations. First, cytotoxicity assessments were performed only at 24 and 48 h, and no long-lasting effects were observed. Furthermore, biocompatibility based on material content was assessed, but no quantitative analysis of specific monomers or byproducts was performed. The in vitro model used, although it provides controlled conditions, does not fully reflect the dynamic characteristics of the oral environment, such as saliva flow, microbial colonization, mechanical forces, and host immune response. Another limitation of this study was the use of a control group. In this study, PBS was used only as an additional reference group to demonstrate the effect of a non-nutritive solution and could not be considered a true positive control. This methodological limitation must be considered when interpreting our findings. Finally, the exclusion of surface polishing, aging protocols, and light polymerization times from the evaluation partially limited the direct translation of the results to the clinical setting. Therefore, long-term cytotoxicity studies, multifaceted studies supported by monomer release analyses, clinical follow-up data, and studies evaluating different parameters are needed.

The data obtained with *in-vitro* study design provide guidance for clinical studies. In this study, the cytotoxic effects of these materials were evaluated, with limited studies in the literature. Among the materials evaluated, the least toxic material was Glasiosite, and the most toxic material was BEAUTIFIL-Bulk Restorative. We believe that it is important to diversify studies evaluating these material groups, which are important in pediatric dentistry owing to their bioactive characteristics, to guide clinicians in material selection and clinical decision-making.

## Conclusion

This study demonstrated that restorative materials differ significantly in their cytotoxic potential, with Glasiosite being the most favorable and Beautifil Bulk Restorative the least. A critical observation was that the extract method resulted in higher cell viability than the direct contact method, highlighting the strong impact of the exposure type.

## Data Availability

The authors declare that all data generated or analyzed during this study are included in this published article.
